# 

**Published:** 1876-08

**Authors:** 


Vol. XXXIII.
Number 8.

August, 1876.

NEW SERIES.
Vol. I.
Number 8.

THE

### CHICAGO MEDICAL Journal and Examiner

PUBLISHED MONTHLY.

EDITOR:
WILLIAM H. BYFORD, A.M., M.D.

ASSOCIATE EDITORS:

JAMES H. ETHERIDGE, M.D. | JAS. NEVINS HYDE, A.M., M.D.

NORMAN BRIDGE, M.D. | FERD. C. HOTZ, M.D.

Published under the auspices of the

*CHICAGO MEDICAL PRESS ASSOCIATION.*

**TERMS—FOUR DOLLARS PER ANNUM, IN ADVANCE.**
**POSTAGE FREE.**

FOLIA SUA AD SANITATEM GENTIUM

CHICAGO:
W. B. KEEN, COOKE & CO., Publishers,
Nos. 113 and 115 State Street.

